# Case of Traumatic Femoral Aneurism, Treated by Digital Compression.—Ligation Afterwards of the External Iliac Artery

**Published:** 1864-07

**Authors:** Jackson Chambliss

**Affiliations:** Surgeon P. A. C. S.


### CONFEDERATE STATES MEDICAL & SURGICAL JOURNAL.

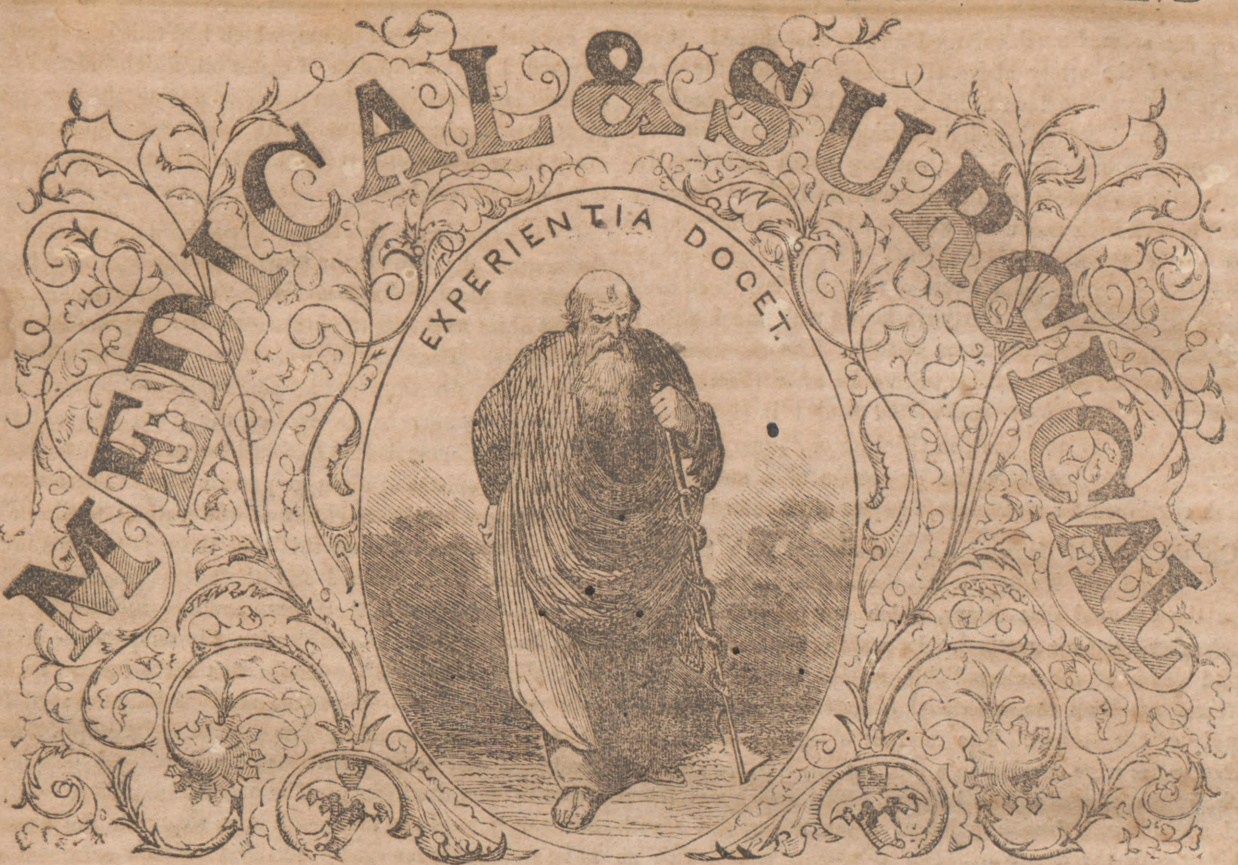

VOL. I. RICHMOND, JULY, 1864. No. 7.

### ORIGINAL COMMUNICATIONS.

ART. I.—*Case of Traumatic Femoral Aneurism—Treated by Digital Compression—Ligation afterwards of the External Iliac Artery.* Reported by JACKSON CHAMBLISS, Surgeon P. A. C. S.

Private John H. Gatewood, company "F," 21st Georgia regiment; aged 32; five feet eleven inches in heighth; weight 145; light hair; blue eyes; fair complexion; occupation a farmer. General health good, when enlisted, on the 24th of January, 1861. Wounded at the second battle of Manassas, August 28th, 1862. General health good previous to and at the time of receiving wound. Small ball entering just behind left trochanter, passing out below perineum, entering right thigh opposite, passing through and coming out at the external aspect at middle of upper third of the thigh; bleeding was profuse for several minutes; his clothes were taken off, and cold water poured from a canteen upon his head and limb; hemorrhage was soon arrested, when he was removed to Middleburg, Virginia, where he remained nine weeks closely confined to bed. His health during this time was, in other respects, good. He was now sent home, where he remained until April 12th, 1863, when he was received as a patient in Polk Hospital, Rome, Georgia. Here digital compression was used for fifty-three hours; pulsation was not arrested in the tumor after removing the compression. From irritation of the thumb, a large bubo resulted in the groin, which gave much uneasiness for three or four weeks.

Patient entered the hospital October 31st, 1863. His condition was as follows: General health good; complained only of right thigh and leg; could walk about some; but the least exertion of body or mental excitement produced great pain, which was principally located in the region of the wound at upper third of thigh; the thigh was much enlarged at upper third; there was an extensive bulging of the tissues internally, which extended quite to the perineum; there was also a bulging of the parts in the course of the femoral artery for five or six inches, extending to near the ramus of the pubis. The *bruit* could be plainly heard in this part of the tumor at all times; it could not be heard so plainly internally, or over the largest part of the tumor, except after exercise. There was no general discoloration of the skin over the tumor. At the point where digital compression was used over the ramus of the pubis there was a dark-bluish discoloration. The cicatrix, which was in the centre of the discoloration, was of even a darker hue, and very sensitive to the touch. The femoral artery at this point seemed much enlarged and funnel-shaped. Compression of the artery above the tumor arrested the *bruit.* The patient was confined to his ward and his diet regulated. On the 15th of December, digital compression was again used for one and a half hours; but, causing much pain, was discontinued; no benefit resulting. On the 18th of January, 1864, compression was used, with bandage applied from the toes up; compresses being applied over the region of the tumor and up to the point where compression had been used. This was continued for thirty-six hours, with much pain to the patient, and resulting in no benefit. The aneurism first made its appearance in about two weeks after the reception of the wound. The external wound healed in eight weeks. It may be well to state, that, on the 15th of January, constant hemorrhage resulted from the extraction of a molar tooth, which continued for eight days, in spite of all remedies used.

On the 31st of January, in consultation with Surgeons Gibson, Read and Dudley, it was decided as best to ligate at once the external iliac artery, which was performed pretty much after Cooper's method. In making the incision down to the tendon of the external oblique muscle, care was taken to tear through all the superficial epigastric vessels with the handle of the scalpel. The internal oblique and transversalis muscles were divided on a grooved director, for about an inch, in the course of the incision. The artery was found and slightly raised with point of index finger, and sheath divided with the finger nail, and a ligature passed with no difficulty. Previous to tying the ligature, the artery was compressed between the thumb and finger, which promptly controlled all pulsation in the tumor. There was hardly an ounce of blood lost in the operation; the lips of the wound were brought together with four or five interrupted sutures, supported by adhesive straps; compresses of lint were applied with a roller bandage; the limb was enveloped in cotton batting, and bandaged from the toes up; the patient was placed in bed, with thigh and leg slightly flexed, the knee being supported on a pillow. Chloroform was administered during the operation; from the effects of which the patient re-acted well.

On the evening of the 31st instant, ten hours after the operation, I found the patient restless and complaining of some pain in the wound. There had been a slight discharge of blood, which had coagulated, adhering the compresses closely to the wound. Pulse 96. Respiration 18, easy. Temperature of hand 76°, affected foot 60°, opposite foot 68°, Fahrenheit. Skin warm and moist. Had had two full consistent dark-colored evacuations immediately after the operation, causing no pain. Opiates administered to induce sleep. There was but little change in his condition until February 3d, when a dark discoloration appeared around the lower lip of wound, resembling ecchymosis. From this an erysipelatous blush extended over the scrotum and crest of ilium, up the right side, to near the seventh rib. Pulse 120. Respiration 25. Temperature of hand 96°, affected foot 86°, opposite foot 89°. Skin soft and dry. Tongue furred in middle, tips and edges red. Having given, on the previous night a dose of calomel, ipecac, and opium, which had failed to operate as directed, I gave a full dose of castor oil, which induced three copious and dark-colored evacuations during the day. The parts were thoroughly painted with tincture of iodine, repeated every three hours, and tr. ferri. chlor., thirty drops, administered every three hours.

On the evening of the 4th, all dressings were removed from the wound, the lips of the same appearing entirely healed, except at point of ligature about the centre, the upper lip looked quite well, and the lower somewhat improved from the day before. There was a thin sanious discharge which was quite fœtid; dressings of lint were continued, being retained in place by roller bandages, and the parts frequently wet with disinfectants; iodine having blistered the parts, was discontinued; opiates at night to procure rest. There was but little change in the condition of the patient until the 9th inst., except that during this time the erysipelatous inflammation had been subdued and the tongue cleaned off. On the evening of the 9th there was a profuse grumous discharge, very fœtid, pulse 114, respiration 21, temperature of hand 98°, affected foot 88°, opposite foot 90°; tongue healthy; skin warm and moist; patient much debilitated; tr. ferri. chlor. decreased to thirty drops, with quin. sulph. 2 grains ter-daily; diet more nutritious; an occasional dose of oil given to open the bowels. The grumous discharge continued incessantly until the fourteenth day, requiring the dressings to be frequantly renewed. The lint was now saturated with tr. ferri. chlor., and disinfectants were also freely used.

On the 15th inst. all discharge ceased, the clots of blood came away from the wound which presented a healthy granulated appearance, with discharge of healthy pus. The appetite, which was at first poor, had now greatly improved. The spirits of the patient good, and his whole condition flattering; pulse 110, respiration 17, temperature of hand 88°, affected foot 78°, opposite foot 80°. He now continued to improve until the afternoon of the 17th inst., the eighteenth day after the operation, when violent arterial hemorrhage occurred, from which he died in a few minutes I saw him immediately after the bleeding commenced, he was then speechless, pulseless, and bathed in a cold perspiration. No remedies could be administered. On examination, I found the wound and bed flooded with blood.

*Post Mortem Appearances.*—External wound gaping and filled with clotted blood; lower lip much sloughed; ulceration over artery where digital compression was used; ligature detached with some effort, holding a firm clot in its tie; blood extravasated extensively under the integuments over crest of ilium, and up the corresponding side to near the seventh rib; tissues were blackened and filled with clotted blood and pus in the course of the erysipelatous inflammation. A careful dissection of the thigh revealed two aneurismal tumors, one 5¼ inches in length and 3¼ inches in diameter, and the other 3 inches in length and 1⅝ inches in diameter, each of an ovoid form and connected together by a neck ½ an inch in length and ⅝ inch in diameter, freely communicating the two cavities; through this neck entered the profunda and vein, side by side.

The largest tumor laid internally imbedded in the fibres of the adductor brevis muscle, and perfectly enveloped by them, and extending nearly the whole length of this muscle—a deep hollow or bed being formed in the surrounding muscle corresponding to the size of the tumor. This tumor is seemingly formed by the expansion of the vein. The smaller tumor laid immediately under the femoral artery, extending some distance above and below the origin of the profunda artery, and resting upon the inner border of the vastus interum muscle, and upon the thigh bone. The portion of this muscle beneath the tumor was almost entirely absorbed, making a deep impression, which extended quite to the bone. This tumor was seemingly formed by the expansion of the artery. An incision made into the larger tumor revealed slightly clotted dark blood, with no fibrinous deposits or layers; the sac, or membrane proper, was about double the thickness of the femoral artery, but much more dense and tenacious in its structure; internal surface smooth, resembling a serous surface, of a pale, pinkish color, with here and there dark clouded discolorations. The small tumor contained dark blood unclotted, the membrane of same, and the neck connecting the two tumors being of the same thickness of the larger tumor; but two openings exist in these tumors or the neck. The entrance of the profunda artery forming one, and that of the profunda vein the other. These vessels are quite small at their junction with the neck of the tumors; smaller than at their origins about one inch above. On examination of external iliac artery at point of ligature, the upper end was found completely plugged for one and a quarter inches, and the lower end imperfectly for one inch, this plug being apparently washed out by the recurrent circulation. The external iliac gives off neither the epigastric nor circumflex arteries, these vessels arising in common with other small vessels from large trunks given off from the femoral, just below Poupart's ligament. One of these trunks, half the size of the femoral, coursed downwards for several inches, with the femoral lying close by its side, previous to dividing into small vessels. The femoral artery and vein were found perfectly healthy, and of natural size throughout their course. The wound through the abdominal muscles was perfectly healthy. The external iliac artery was slightly adherent to the vein at point of ligature, the vein, however, was perfectly healthy and pervious. The peritoneum was found perfectly healthy, with no tendency to protusion of the intestines. There was no extravasation of blood within pelvic cavity or abdominal muscles previous to performing the operation of ligation of the external iliac. I saw my patient almost daily for three months, never failing to examine closely the condition of the tumor. The diagnosis, to my mind, of the diseased condition of the femoral was beyond doubt, and I was perfectly satisfied that it was greatly enlarged at the point where digital compression was used. Out of a number of professional gentlemen who examined the case with me, I know of not one who differed from me in the above opinion. The internal bulging was more mysterious. We were not satisfied as to whether the profunda was implicated or not. The apparent funnel shape of the femoral at the point where digital compression was used can, I think, be accounted for when we consider the large trunk which laid by its side, instead of feeling one artery, the impulse of two was given to the touch. It was impossible to trace the pulsations of the femoral over the small tumor; this I tried repeatedly.

The sutures were all removed on the 5th of February, having ulcerated but slightly; the sanious discharge contained many fat, but few pus globules; there was a slight *bruit* detected in the tumor on the 6th inst., which continued until the death of the patient with but little increase; no pulsation could be detected in the arteries of the popliteal space, leg or foot; the toes and patella of the limb were cooler to the touch than the surface of the limb, or the toes and patella of the opposite limb, yet the patient did not complain of the least uneasiness in the limb from the first; the limb at no time seemed to require any degree of artificial heat. The tumor seemed to decrease greatly after the first few days of the operation; the lips of the wound, though apparently healed, were soon broken down by the grumous discharge, causing some ulceration. The ligature securing the artery was but little interfered with during the treatment. There was considerable nervous excitement until the tenth day, requiring frequent and liberal doses of opium to procure rest; the patient was, however, quite rational and resolute; the tongue was never heavily coated; there was more or less thirst until the fourteenth day. Respiration, though frequent, was always easy; the bowels, though constipated, were not obstinately so; there was not the least tendency to peritonitis; the skin was usually warm and moist, without profuse perspiration; the urine was generally scant and highly colored, and passed with some pain,; there were no marked deposits; the pulse, though frequent and full, was quite compressible.

From tabulated notes, taken morning and night, daily, and filed with this report in the office of the Surgeon-General, I have prepared the following summary:

| From evening of Jan. 31st to evening of Feb. 15th. | Evening. | Average. | Morning. | Average. | Total average of all reckonings taken. |
|---|---|---|---|---|---|
| Pulse ranged from | 96 to 130 | 119 | 90 to 130 | 115 | 117 |
| Respiration ranged from . . . . . | 18 to 30 | 24 | 16 to 29 | 22 | 23 |
| Temperature of hand ranged from | 76° to 99° | 93° | 88° to 99° | 96° | 94° |
| Temperature of affected foot ranged from . . . . . | 60° to 88° | 82° | 66° to 91° | 84° | 83° |
| Temperature of opposite foot ranged from . . . . . . | 68° to 93° | 85° | 68° to 94° | 87° | 86° |

